# ATF4 Involvement in TLR4 and LOX-1-Induced Host Inflammatory Response to *Aspergillus fumigatus* Keratitis

**DOI:** 10.1155/2018/5830202

**Published:** 2018-12-13

**Authors:** Shuchao Zhang, Pingping Meng, Guibo Liu, Kuixiang Liu, Chengye Che

**Affiliations:** ^1^Department of Blood Transfusion, The Affiliated Hospital of Qingdao University, Qingdao, China; ^2^Department of Physical Medicine and Rehabiliation, The Affiliated Hospital of Qingdao University, Qingdao, China; ^3^Department of Ophthalmology, The Affiliated Hospital of Qingdao University, Qingdao, China; ^4^Department of Ophthalmology, The Eighth People's Hospital of Qingdao, Qingdao, China

## Abstract

**Purpose:**

Activating transcription factor 4 (ATF4) is induced by various stressors. Here, we investigated the expression of ATF4 in the host inflammatory response to *Aspergillus fumigatus* (*A. fumigatus*) keratitis.

**Methods:**

*A. fumigatus* keratitis mouse models developed by intrastromal injection as well as corneal epithelium scratching were examined daily with a slit lamp microscope for corneal opacification and ulceration. Subsequent *in vitro* experimentation was carried out in human corneal epithelial cells (HCECs) as well as THP-1 macrophages infected with *A. fumigatus*. Inhibitors, including CLI-095, Poly (I), SCH772984, and SP600125, were used to assess the role of proteins like toll-like receptor 4 (TLR4), lectin-type oxidized LDL receptor 1 (LOX-1), extracellular signal-regulated kinases (ERK1/2), and c-Jun N-terminal kinase (JNK) in ATF4 expression as a response to *A. fumigatus* infection. This assessment was made in both mouse models and HCECs using western blot.

**Results:**

Compared to the controls, ATF4 was increased in corneas from two kinds of *A. fumigatus* keratitis models at 3 days after infection. ATF4 expression was upregulated with *A. fumigatus* conidia both in HCECs and THP-1 macrophages 16 hours after stimulation. Furthermore, ATF4 expression in response to *A. fumigatus* infection was shown to be dependent on TLR4 and LOX-1 expression, and ERK1/2 and JNK contributed to the expression of ATF4 in response to *A. fumigatus*.

**Conclusion:**

Our results clearly indicate that ATF4 was involved in the host antifungal immune response to *A. fumigatus* keratitis; expression was found to be dependent on TLR4, LOX-1 expression, and MAPKs pathway.

## 1. Introduction

As severe infectious corneal diseases are mostly caused by *Fusarium solani* and *Aspergillus fumigatus*, fungal keratitis is likely to damage visual acuity and cause blindness [1–3]. Separate from *Acanthamoeba* keratitis, the typical clinical features of fungal keratitis are difficult to ascertain in the early stage of infection, leading to common misdiagnoses and delayed treatment [4, 5]. Although there are new therapies used in clinical application, fungal keratitis remains a challenge to ophthalmologists due to a lack of effective drugs after late initiation of treatment secondary to delayed diagnosis [6, 7].

Innate immunity is the first line of defense against infection by pathogenic fungi during fungal keratitis. Pattern recognition receptors (PRRs), including toll-like receptor 2 (TLR2) [8, 9], toll-like receptor 4 (TLR4) [10, 11], lectin-type oxidized LDL receptor 1 (LOX-1) [11, 12], and dendritic cell-associated c-type lectin-1 (dectin-1) [10, 13], each play an important role in antifungal immunity. Previous studies have found that the TLR4/MyD88 pathway, induced by lipopolysaccharide (LPS) activation and translocation, activated transcription factor 4 (ATF4) in the nucleus in human monocytes [14]. In addition, TLR2 expression was increased by ATF4 upregulation as part of endoplasmic reticulum (ER) stress. This upregulation was found to induce the production of cathelicidin and KLK5 so as to mediate the activation of proinflammatory and antimicrobial immune responses [15].

ATF4, also known as tax-responsive enhancer element B67, is a protein encoded by the ATF4 gene in human. ATF4 expression is induced by ER as well as oxidative stress and is upregulated by eukaryotic initiation factor 2 phosphorylation in the protein kinase R-like ER kinase pathway. ATF4 has also been shown to be involved in the TLR-mediated innate immune response [14, 15]. However, the expression of ATF4 in fungal keratitis has not been previously studied. To this end, we executed *in vivo* and *in vitro* experiments in mouse models, human corneal epithelial cells (HCECs), and THP-1 macrophages infected with *A. fumigatus* to fill this knowledge gap. We were able to confirm that ATF4 was involved in host antifungal immune response in *A. fumigatus* keratitis and was dependent on TLR4, LOX-1 expression, and MAPKs pathway.

## 2. Methods

### 2.1. Preparation of *A. fumigatus*

*A. fumigatus* strain 3.0772 was purchased from the China General Microbiological Culture Collection Center (Beijing, China) and was cultured for 3-4 days on Sabouraud agar. Suspensions of fresh conidia scraped from the medium surface were quantified using a haemocytometer and adjusted to a final concentration of 5 × 10^4^ conidia/*µ*L in phosphate-buffered saline (PBS).

### 2.2. Keratitis Mouse Model Developed by Intrastromal Injection

Eight-week-old C57BL/6 female mice were purchased from the Changzhou Cavens Laboratory (Jiangsu, China). Experimental mice were treated in accordance with the *Statement for the Use of Animals in Ophthalmic and Vision Research* by the Association for Research in Vision and Ophthalmology (ARVO). Mice were anaesthetized with 8% chloral hydrate, and then one eye was randomly selected from each mouse. *A. fumigatus* conidia (2 *µ*L, concentration 0.5 × 10^5^ *µ*L) were released into the corneal stroma of the mouse [16], and the mice were subsequently examined by a slit lamp microscope for corneal opacification and ulceration. Corneas were harvested for use in western blot assay at 1/2, 1, 2, 3, 5, 7, 10, and 14 days after infection.

### 2.3. Keratitis Model Developed by Corneal Epithelium Scratch

One eye was randomly selected from one mouse. The central corneas were scratched (making a three 1 mm incision) using a sterile 25^5/8^ gauge needle, and then the scratches were covered with *A. fumigatus* conidia (5 *µ*L, concentration 1 × 10^8^ *µ*L). The eyelid was then sutured with a soft contact lens to cover the corneal surface [11]. Mice were examined by a slit lamp microscope, and corneas were harvested for western blot assay at 1, 3, and 5 days after infection.

### 2.4. Culture of HCECs

Human corneal epithelial cells (HCECs) were provided by the Ocular Surface Laboratory at the Zhongshan Ophthalmic Center. Cells were cultured to 80% confluence in serum-free DMEM (HyClone, USA) for 24 hours and then treated in 6-well plates with 100 ng/mL lipopolysaccharide (LPS) (Sigma-Aldrich, USA) [17], 5 *µ*g/mL *β*-1, 3-glucan (Santa Cruz, USA) [18], 1 ng/mL transforming growth factor beta (TGF-*β*) (ProSpec, Israel) [19], 10 ng/mL interleukin 1 beta (IL-1*β*) (ProSpec, Israel) [19], and *A. fumigatus* conidia at a multiplicity of infection (MOI) of 1 for 8 and 16 hours. These cells were then used in western blot assay.

### 2.5. Culture of THP-1 Macrophages

THP-1 macrophages were purchased from the China Center for Type Culture Collection (Wuhan, China) and differentiated with 100 nM of phorbol 12-myristate 13-acetate (PMA) (Sigma-Aldrich, USA) for 48 hours then allowed to recover for 24 hours prior to infection. Macrophages were cultured in RPMI-1640 medium at a density of 1 × 10^6^ mL and then were treated in 6-well plates with 100 ng/mL LPS, 5 *µ*g/mL *β*-1, 3-glucan, 1 ng/mL TGF-*β*, 10 ng/mL IL-1*β*, and finally *A. fumigatus* conidia at a MOI of 1 for 8 and 16 hours. Theses samples were then analyzed with western blot assay.

### 2.6. ATF4 Expression Pathway Experiments

Mouse models developed by intrastromal injection and HCECs were selected for ATF4 expression pathway experiments. To assess the function of TLR4, LOX-1, ERK1/2, and JNK in reaction to ATF4 expression upon *A. fumigatus* infection, randomly selected eye from the mouse model received a subconjunctival injection (5 *µ*L) containing 0.5 *μ*g CLI-095 (InvivoGen, USA), 2 *μ*g Poly (I) (Sigma-Aldrich, USA), 40 *μ*M SP600125 (SelleckChem, USA), or 10 *μ*M SCH772984 (SelleckChem, USA) 1 day and 2 hours before infection [11, 16]. DMSO or sterile water was used as control, respectively. *A. fumigatus* conidia (2 *µ*L, concentration 0.5 × 10^5^ *µ*L) were released into corneal stroma of the mouse models, and then corneas were harvested for western blot analysis 3 days after infection. In regards to *in vitro* experiments, HCECs were pretreated with 1 *μ*M CLI-095 [20], 250 *μ*g/ml Poly (I) [21], 40 *μ*M SP600125, or 10 *μ*M SCH772984 2 hours before conidia treatment. DMSO or sterile water was used as controls, respectively. After pretreatment, *A. fumigatus* conidia was introduced into HCECs culture at a MOI of 1 for 16 hours, and then cells were harvested for western blot assay.

### 2.7. Western Blot Assay

The western blot assay protocol used for analyzing corneas and cell samples has been described in previous publications [12, 16]. Membranes were incubated accordingly with anti-ATF4 (ProteinTech, USA) and anti-*β*-actin (CST, USA). The HRP-tagged secondary antibodies used were purchased from CST. The Fusion Solo system (VilberLourmat, France) developed blots with ECL substrate; additionally, histograms were used to quantify and represent chemiluminescence.

### 2.8. Statistical Analysis

An unpaired two-tailed Student's *t*-test was used to determine the statistical significance of western blot data and was represented as mean ± SD and was analyzed using GraphPad 5.0 software. Data were considered significant at *P* ≤ 0.05.

## 3. Results

### 3.1. ATF4 Was Increased in Mouse Corneas Infected by Intrastromal Injection

**Figure 1:**
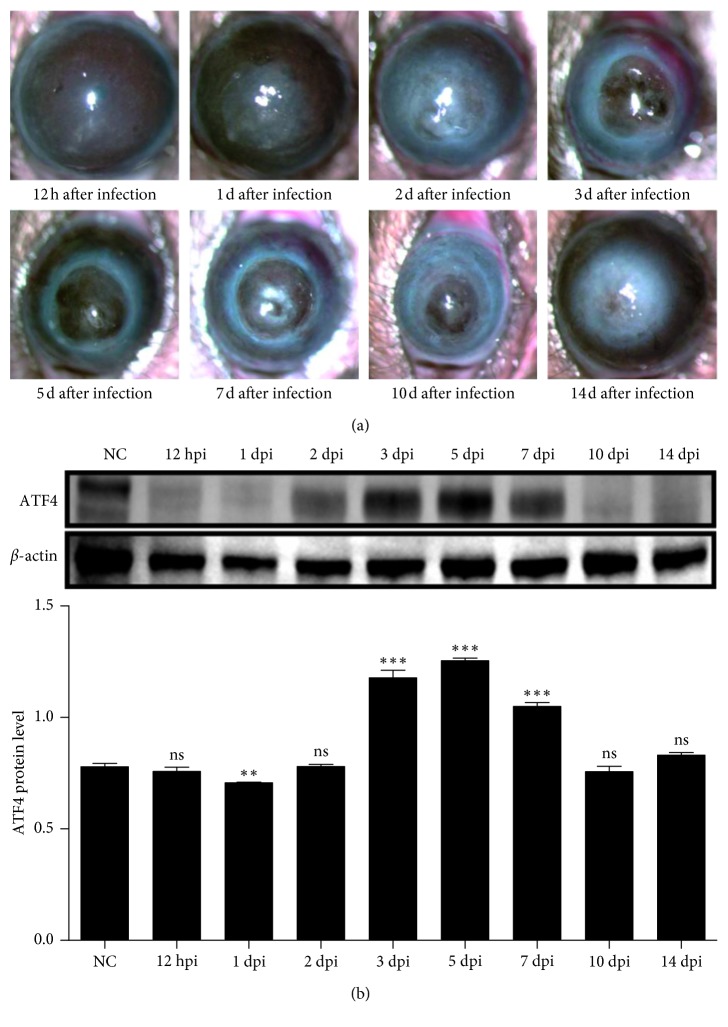
ATF4 was increased in mice corneas of *A. fumigatus* keratitis developed by intrastromal injection. (a) Significant corneal opacity of C57BL/6 mouse models was developed by intrastromal injection formed 1 day after infection and persisted up to 14 days. Corneal inflammation gradually improved with increased neovascularization. (b) After *A. fumigatus* infection, ATF4 protein expression was confirmed by western blot to be degraded in mouse corneas at 1 day after infection, elevated at 3 days, and then persisted up to 7 days. As the inflammation subsided, ATF4 expression returned to normal after 10 days. Mean values and standard deviations of two independent experiments are shown; ^*∗∗*^*P* < 0.01, ^*∗∗∗*^*P* < 0.001.

Significant corneal opacity developed in C57BL/6 mouse models at 1 day after infection via intrastromal injection. This opacity persisted up to 14 days (Figure 1(a)) but corneal inflammation gradually improved with increased neovascularization. Compared with controls, ATF4 protein was degraded in infected mouse corneas at 1 day after infection (*P* < 0.05), then elevated at 3 days, and persisted up to 7 days (*P* < 0.05). As inflammation subsided, the expression of ATF4 returned to normal 10 days after infection (*P* > 0.05) (Figure 1(b)).

### 3.2. ATF4 Increased in Mouse Corneas Infected by Corneal Epithelium Scratch

**Figure 2:**
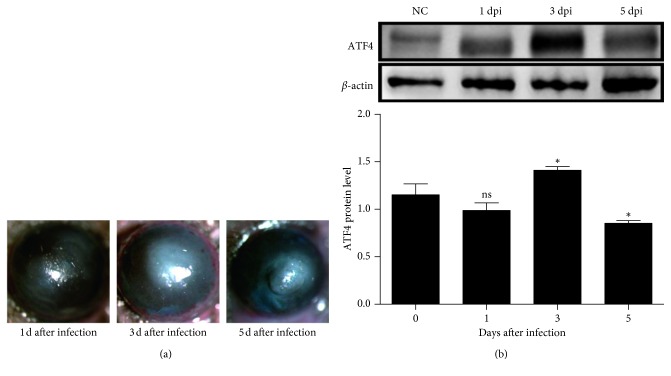
ATF4 was increased in mice corneas of *A. fumigatus* keratitis developed by corneal epithelium scratch. (a) The significant corneal opacity of C57BL/6 mice models was developed by corneal epithelium scratch formed at 1 day after infection, which persisted up to 5 days. (b) After *A. fumigatus* infection, ATF4 protein expression was confirmed by western blot to be elevated in infected mouse corneas at 3 days after infection and completely degraded at 5 days. Mean values and standard deviations of two independent experiments are shown; ^*∗*^*P* < 0.05.

The significant corneal opacity of C57BL/6 mice models developed by corneal epithelium scratch formed 1 day after infection and persisted up to 5 days (Figure 2(a)). Compared with controls, ATF4 protein levels were elevated in infected mouse corneas at 3 days after infection (*P* < 0.05) and then started to degrade by 5 days (*P* < 0.05) (Figure 2(b)).

### 3.3. ATF4 Increased Amount of Stimulated HCECs and THP-1 Macrophages in Response to *A. fumigatus*

**Figure 3:**
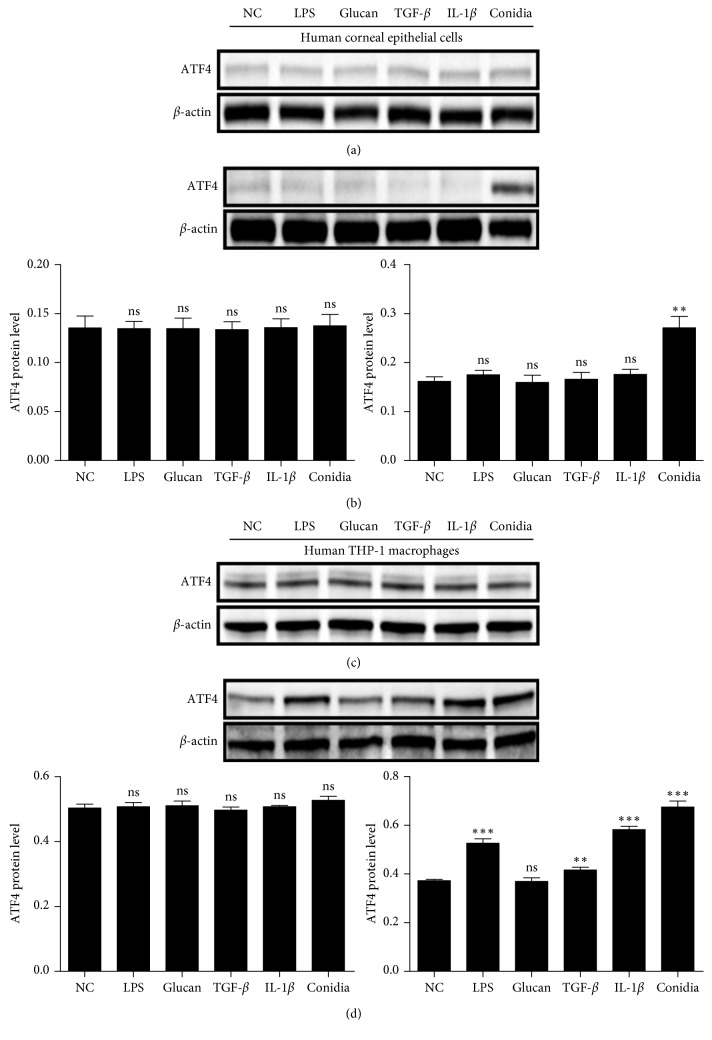
Increased expression of ATF4-stimulated HCECs and THP-1 macrophages. (a) With the stimulation of LPS, *β*-1, 3-glucan, TGF-*β*, IL-1*β*, and *A. fumigatus* conidia, ATF4 protein levels in HCECs were not changed significantly at 8 hours. (b) ATF4 protein levels in HCECs did not change significantly at 16 hours with the stimulation of LPS, *β*-1, 3-glucan, TGF-*β*, and IL-1*β*, but significantly increased with *A. fumigatus* conidia. (c) With the stimulation of LPS, *β*-1, 3-glucan, TGF-*β*, IL-1*β*, and *A. fumigatus* conidia, ATF4 protein levels in THP-1 macrophages did not change significantly at 8 hours. (d) ATF4 protein levels in HCECs were higher after stimulation with LPS, TGF-*β*, IL-1*β*, and *A. fumigatus* conidia at 16 hours, but did not change significantly with *β*-1, 3-glucan. The mean values and standard deviations of two independent experiments are shown; ^*∗∗*^*P* < 0.01, ^*∗∗∗*^*P* < 0.001.

With stimulation by LPS, *β*-1, 3-glucan, TGF-*β*, IL-1*β*, and *A. fumigatus* conidia, ATF4 protein levels in HCECs did not change significantly at 8 hours (*P* > 0.05, respectively) (Figure 3(a)). Additionally, ATF4 protein levels in HCECs did not change significantly at 16 hours (*P* > 0.05, respectively) with LPS, *β*-1, 3-glucan, TGF-*β*, and IL-1*β* stimulation. Interestingly, ATF4 protein levels were significantly increased with the addition of *A. fumigatus* conidia (*P* < 0.05) (Figure 3(b)).

With the stimulation of LPS, *β*-1, 3-glucan, TGF-*β*, IL-1*β*, and *A. fumigatus* conidia, ATF4 protein levels in THP-1 macrophages were not changed significantly at 8 hours after stimulation (*P* > 0.05, respectively) (Figure 3(c)). Conversely, ATF4 protein levels in THP-1 macrophages were elevated after stimulation with LPS, TGF-*β*, IL-1*β*, and *A. fumigatus* conidia at 16 hours (*P* < 0.05, respectively), but did not change significantly with *β*-1, 3-glucan stimulation (*P* > 0.05) (Figure 3(d)).

### 3.4. ATF4 Expression in Response to *A. fumigatus* Infection was Dependent on TLR4 and LOX-1

**Figure 4:**
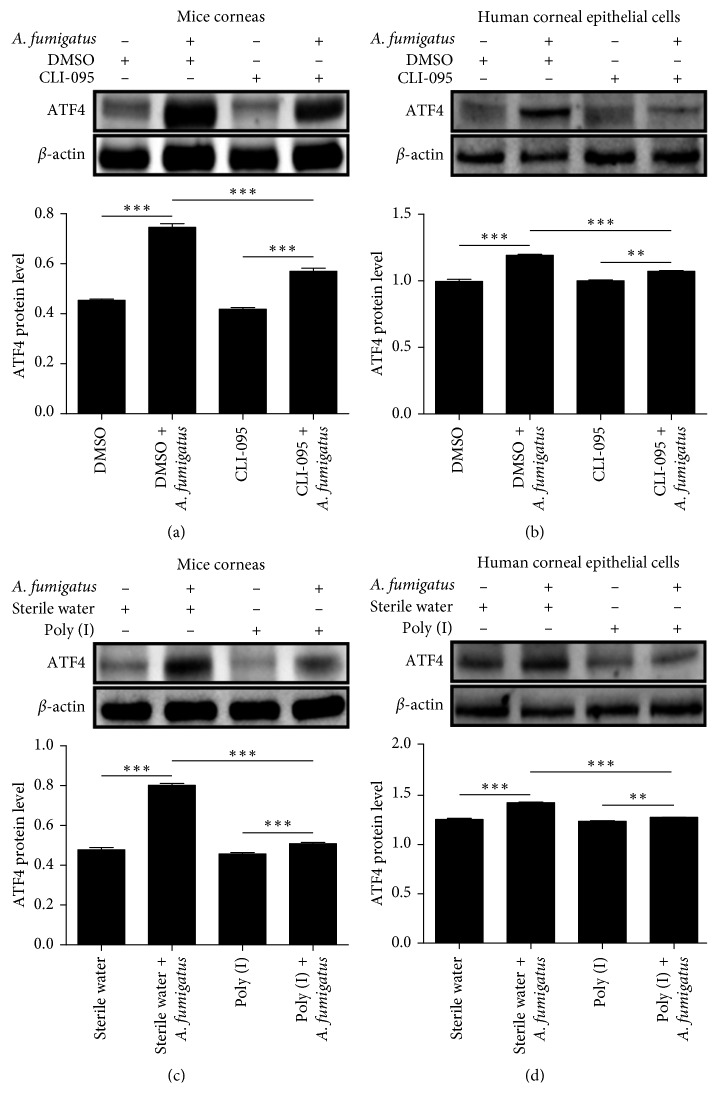
ATF4 expression in response to *A. fumigatus* infection was dependent on TLR4 and LOX-1. With pretreatment using a TLR4 inhibitor, ATF4 protein levels in infected mouse corneas (a) at 1 day after infection and HCECs (b) at 16 hours after *A. fumigatus* infection were significantly lower. ATF4 protein levels in infected mouse corneas (c) and HCECs (d) were significantly lower with the pretreatment of LOX-1 inhibitor. The mean values and standard deviations of two independent experiments are shown; ^*∗∗*^*P* < 0.01, ^*∗∗∗*^*P* < 0.001.

With TLR4 inhibitor pretreatment, ATF4 protein levels in infected mouse corneas (Figure 4(a)) at 1 day after infection and HCECs (Figure 4(b)) at 16 hours after *A. fumigatus* infection were significantly suppressed (*P* < 0.05, respectively). Similarly, ATF4 protein levels in infected mouse corneas (Figure 4(c)) and HCECs (Figure 4(d)) were significantly lower (*P* < 0.05, respectively) with pretreatment of a LOX-1 inhibitor.

### 3.5. ERK1/2 and JNK Contributed to Expression of ATF4 in Response to *A. fumigatus*

**Figure 5:**
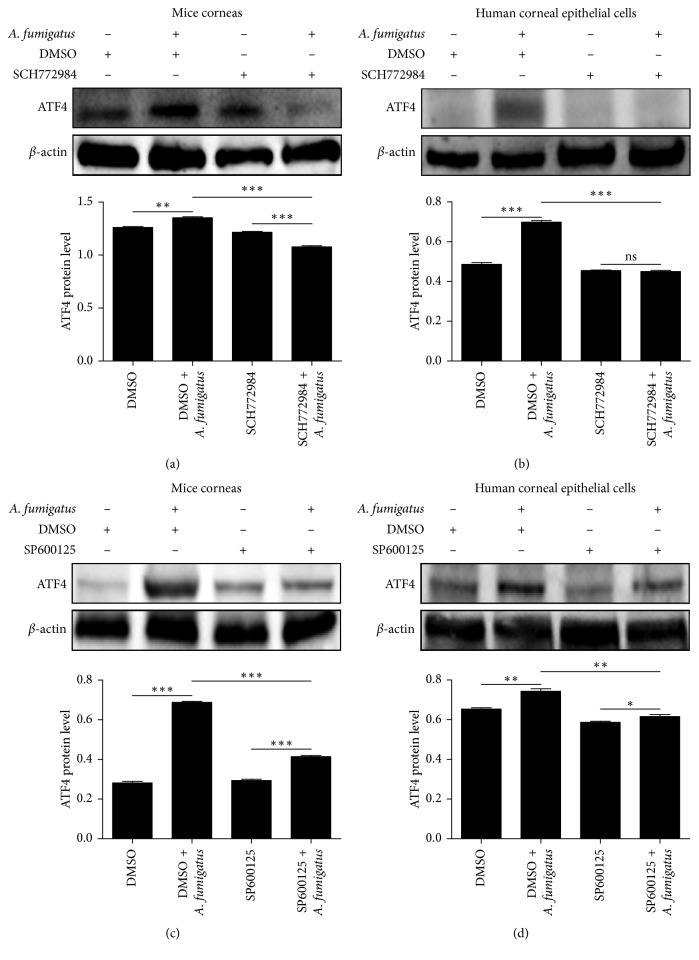
ERK1/2 and JNK contributed to expression of ATF4 in response to *A. fumigatus.* With pretreatment of an ERK1/2 inhibitor, ATF4 protein levels in infected mouse corneas (a) 1 day after infection and HCECs (b) 16 hours after *A. fumigatus* infection were significantly lower. ATF4 protein levels in infected mouse corneas (c) and HCECs (d) are significantly lower with pretreatment by JNK inhibitors. The mean values and standard deviations of two independent experiments are shown; ^*∗*^*P* < 0.05, ^*∗∗*^*P* < 0.01, ^*∗∗∗*^*P* < 0.001.

With ERK1/2 inhibitor pretreatment, ATF4 protein levels in infected mouse corneas (Figure 5(a)) at 1 day and HCECs (Figure 5(b)) at 16 hours after *A. fumigatus* infection were significantly attenuated (*P* < 0.05, respectively). Likewise, ATF4 protein levels in infected mouse corneas (Figure 5(c)) and HCECs (Figure 5(d)) were significantly lower (*P* < 0.05, respectively) with pretreatment using a JNK inhibitor.

## 4. Discussion

Our results demonstrate that ATF4 was involved in the host antifungal immune response to *A. fumigatus* keratitis. This involvement was dependent on expression of TLR4, LOX-1, ERK1/2, and the JNK pathway. Although the role of ATF4 in fungal keratitis is still not well understood, many studies have provided support to its role in inflammation. For example, Woo et al. [22] demonstrated that ATF4 mediated the upregulation of rottlerin-induced COX-2 via the p38 mitogen-activated protein kinase (MAPK) pathway. Then, Hayashi et al. [23] examined ATF4 and found it suppressed activation of T cells by inhibiting L-type amino acid transporter 1 and increasing homeobox B9 expression induced by amino acid deprivation. Furthermore, Yang et al. [24] showed mediation by the EIF2AK4/GCN2-EIF2S1/eIF2*α*-ATF4 pathway in *Pseudomonas aeruginosa*-induced autophagy-enhanced bacterium fitness in human airways. Taken together, these observations are consistent with our results that ATF4 was involved in host immune response to an infectious disease.

Importantly, ATF4 levels were increased in corneas, compared to controls in mouse *A. fumigatus* keratitis models developed by intrastromal injection or corneal epithelium scratch 3 days after infection. Being the most commonly used mouse models for fungal keratitis research, both models showed similar ATF4 expression trends. As inflammation subsided, the expression of ATF4 returned to normal. Our findings indicate that as an ER stress-associated transcription factor, ATF4, is involved in the host immune response especially in the most serious stages of *A. fumigatus* keratitis.

Additionally, we confirmed that ATF4 expression was upregulated with the addition of *A. fumigatus* conidia both in HCECs and THP-1 macrophages at 16 hours after stimulation. Fungal keratitis is often caused by ocular trauma resulting from eye irritation by vegetative matter [1, 25]. From an anatomical point of view, the epithelium is one of the earliest corneal structures to come in contact with pathogenic fungi. For this reason, human corneal epithelial cells have been shown to be involved in antifungal immunity by releasing antimicrobial peptides, inflammatory cytokines, and chemokines [26–28]. Neutrophils are then recruited to the cornea to fight against fungal infection [29–31].Our findings additionally indicate that ATF4 was involved in fungal keratitis both with regards to the corneal epithelium and immunocytes.

ATF4 has been shown to be induced by multiple types of stimuli. For example, Sasaki et al. [17] identified ATF4 as a mediator of IL-6 expression induced by LPS in embryonic fibroblast cells of p32 deficient mouse. Hara et al. [32] identified that ATF4 mediated IL-23 p19 mRNA expression induced by LPS in a JNK-dependent manner, which could be inhibited by apomorphine. Our results are consistent with reports that ATF4 expression was upregulated with LPS stimulation in THP-1 macrophages, but not in HCECs.

TGF-*β* is a Treg-specific cytokine [33, 34], and IL-1*β* is a Th1 proinflammatory cytokine [35, 36] previously demonstrated to be correlated with ATF4 expression. For example, Liu et al. [37] showed that ATF4 and other ER stress-associated molecules were markedly upregulated by TGF-*β* stimulation in renal tubular cells. Additionally, it was demonstrated by Verma and Datta [38] that ATF4 and other ER stress-associated molecules were induced by IL-1*β* via JNK in human pancreatic epithelial cells. Our results in this study are consistent with these reports that ATF4 expressions were upregulated with TGF-*β* or IL-1*β* stimulation in THP-1 macrophages, but not in HCECs. The expression of ATF4 in THP-1 macrophages was more sensitive to stimuli than in HCECs.

Here, we observed that the expression of ATF4 upon *A. fumigatus* infection was demonstrated to be TLR4 and LOX-1 dependent. As a toll-like receptor, TLR4 activates an intracellular signaling pathway and induces inflammatory cytokine expression as a part of innate immunity [39, 40]. Yang et al. [41] demonstrated that the p-IRE1*α*/p-JNK/CHOP/GRP78/ATF4 pathway that mediates ER stress was triggered by LPS-induced TLR4 activation enhancing the infiltration of monocytes and macrophages in acute renal failure. As a member of the C-type lectin super family, LOX-1 is a multiligand receptor which binds to activated platelets, apoptotic cells, C-reactive protein, and bacteria [42]. Taken together, these observations indicate that ATF4 expression may be induced by PRRs activation, including toll-like receptors and C-type lectin receptors.

In addition, we observed that ERK1/2 and JNK contributed to ATF4 expression in response to *A. fumigatus* infection. ERK1/2 and JNK are MAPK members which play important roles in the cellular response to proinflammatory cytokines [35, 43–45]. ATF4 phosphorylation was shown by Li et al. [46] to be induced by BMP-2/COX-2/PGE2 signaling pathway as part of the EP4-ERK1/2-RSK2 axis in chondrocytes. Furthermore, Lin et al. [47] demonstrated that ATF4 and other ER stress-associated molecules were abolished by the addition of a JNK inhibitor (SP600125) in protodioscin-treated cervical cancer cells. Consistent with our results, these reports from the literature indicate that ATF4 expression may be dependent on MAPKs activation.

In conclusion, our results demonstrate ATF4 was involved in the host antifungal immune response to *A. fumigatus* keratitis. We also demonstrated that such a response was dependent on the expression of the TLR4, LOX-1, and MAPKs pathway. These findings were supported by a range of experimental models including *A. fumigatus* keratitis mice models and cell line experiments.

## Data Availability

The data used to support the findings of this study are available from the corresponding author upon request.
